# Communication practices and information exchange for caregivers of children with cancer in Pakistan

**DOI:** 10.3389/fonc.2025.1616467

**Published:** 2025-09-09

**Authors:** Dylan E. Graetz, Joseph Wardell, Ambreen Hameed, Afia tul Quanita, Atoofa Najmi, Safwan Ahmad, Muhammad Shafiq, Asma Naheed, Shabnam Munir, Gia Ferrara, Courtney Staples, Yichen Chen, Meenakshi Devidas, Carlos Rodriguez-Galindo, Sima Jeha, Jennifer W. Mack, Syed Ahmer Hamid, Alia Ahmad, Muhammad Rafie Raza

**Affiliations:** ^1^ Department of Global Pediatric Medicine, St. Jude Children’s Research Hospital, Memphis, TN, United States; ^2^ University of Child Health Sciences, Children’s Hospital Lahore, Lahore, Pakistan; ^3^ Department of Pediatric Hematology/Oncology, Indus Hospital & Health Network, Karachi, Pakistan; ^4^ Department of Oncology, Dana Farber Cancer Institute/Boston Children’s Hospital, Boston, MA, United States

**Keywords:** pediatric oncology, global health, communication, patient-centered care, information exchange

## Abstract

**Introduction:**

Information exchange is a core component of communication that has been understudied globally. This study sought to examine eight core functions of communication, including information exchange, among caregivers of children with cancer in Pakistan, a middle-income country with >8,000 new cases of childhood cancer each year.

**Methods:**

A cross-sectional survey was administered to 200 caregivers of children with cancer at two major centers in Pakistan. Surveys contained socio-demographic characteristics and questions related to priorities and experiences for communication and information exchange. Surveys were verbally administered from March-November 2023.

**Results:**

While over 90% of caregivers prioritized all eight functions of pediatric cancer communication, significantly fewer (p<0.001) experienced each function. Caregivers wanted to know likelihood of cure (99.5%) and late effects (97%), but how they wanted to receive information varied. Most caregivers (>90%) understood what type of treatment their children would receive; fewer correctly identified diagnosis (77%), location (81%), or treatment duration (71%). Caregivers of patients with leukemia were more likely to have a complete understanding of their child’s diagnosis and treatment (p<0.0001).

**Conclusion:**

Pakistani caregivers express many of the same communication needs noted in other settings, with similar challenges and larger gaps in care. Interventional work should focus on maximizing human resources, ensuring complete information exchange, and empowering caregivers.

## Introduction

Communication is a core element of patient-centered care. In pediatric oncology, high-quality communication facilitates trust (1, 2), increases hope (3, 4) alleviates distress (5), and enables a supportive relationship between families and clinicians (6). In low- and middle-income countries (LMICs), where 90% of childhood cancer cases occur (7), diagnostic communication is essential for establishing rapport and reducing treatment abandonment, a leading cause of morbidity and mortality (8). Unfortunately, most clinicians in LMICs receive limited communication training (9) and the communication preferences and experiences of families in LMICs have been understudied (10).

A functional model for communication was initially established by investigators in the United States for use in adult oncology (11) and has since been adapted for pediatrics (12) and expanded for application in diverse settings (13). The current model includes eight key functions: information exchange, decision making, managing uncertainty, enabling family self-efficacy, building relationships, supporting hope, providing validation, and responding to emotions. In this model, information exchange refers to the bidirectional process in which clinicians communicate important information regarding cause, diagnosis, treatment, prognosis, and late effects, while families share pre-existing beliefs and knowledge, ongoing understanding, and vital information about their child. High-quality information exchange is valued by pediatric oncology patients and families in diverse settings, however prior work has demonstrated gaps in caregiver reported experiences (14, 15). Additionally, values and practices for information exchange vary based on cultural context and resource constraints, including physical space and available interprofessional providers.

In this study, we sought to examine the eight functions of communication and information exchange preferences and experiences of caregivers of children with cancer at two hospitals in Pakistan. We explored caregiver perceptions and conducted an assessment of their understanding to identify opportunities for improvement and potential populations at risk for poor information exchange.

## Materials and methods

### Setting and participants

An estimated 8,000-12,000 children in Pakistan develop cancer each year, where survival is <50% (16). This study was conducted at the two largest pediatric oncology centers in Pakistan, Children’s Hospital of Lahore and Indus Hospital in Karachi. Both are leading institutions for the Pakistani Society of Pediatric Oncology, one of the most active and well-organized pediatric networks in the region and are referral centers for surrounding provinces. Each hospital treats 1000–1500 new pediatric cancer cases per year. Caregivers of newly diagnosed (within 8 weeks) children with cancer (less than age 19) were eligible for the study and were approached sequentially for participation. Signed informed consent was obtained prior to survey administration.

### Survey development and data collection

This study utilized a cross-sectional survey that has been used to assess pediatric cancer communication in other middle-income countries (14, 17) and includes items initially established and validated in US populations (15, 18, 19) with a visual aid for Likert scale questions (17). The English survey was reviewed by Pakistani members of the study team who adapted and added questions for the Pakistani population, as noted below. The survey, including Likert scale anchors, was translated to Urdu and Pashto. Face and content validity were established through cognitive debriefing with 81 caregivers between the two institutions, interviewed over the course of 5 rounds with iterative revision throughout. Once the survey was performing well, it was back translated to English to ensure the intent of the questions was preserved. The final survey was verbally administered from March-November 2023 to 100 caregivers at each site by trained members of the research team.

*Socio-demographic information* was obtained through survey questions on participants’ gender, relationship to the child, language spoken, ethnicity, religion, province, education, and measures of material and financial hardship. Caregivers were also asked about their child’s age and gender. Demographic questions added to the Pakistani version of the survey included residence (urban v. rural) and number of children in the home.

*Communication priorities and experiences* were assessed using validated items (20) structured around eight communication functions previously identified as essential for pediatric cancer communication (12). Caregivers were asked: “How important is it to you that your doctors and other health professionals … explain things in a way I can understand?” (information exchange), “are open and honest with me?” (building relationships), “involve me in making decisions about my child’s care?” (decision-making), “pay attention to my emotions and feelings?” (responding to emotions), “help me deal with the things nobody knows related to my child’s cancer?” (managing uncertainty), “help me understand ways to take care of my child while I’m dealing with cancer?” (self-efficacy), “value my thoughts about my child’s health?” (providing validation), and “provide me with information that makes me hopeful about my child’s cancer and treatment?” (supporting hope). Caregivers responded using a 3-point Likert scale of “very important”, “slightly important”, and “not important” paired with a visual aid utilized in prior work (14, 17). Caregivers’ experiences of each function were assessed through corresponding questions including “how comfortable do your doctors and other health professionals make you feel asking questions?” (information exchange), “how often do your doctors and other health professionals have open and honest communication with you?” (building relationships), “how much do your doctors and other health professionals give you information and resources to help you make decisions about your child’s care?” (decision making), “how well do your doctor and other health professionals talk with you about how to cope with any fears, stress, and other feelings?” (responding to emotions), “how well do doctors and other health professionals help you deal with the things nobody really knows about cancer?” (managing uncertainty), “how often do your doctors and other health professionals make sure you understand the steps in your care?” (self-efficacy), “how often do your doctors and other health professionals help you feel like as a parent you are doing your best”, and “how often are your doctors and other health professionals able to be honest and hopeful at the same time?” (supporting hope). Response options included a 3-point Likert scale. The questions regarding supporting hope and providing validation were adapted from those piloted for an evaluation of these functions in the United States (21). Caregivers were also asked a novel question about trust, a ninth communication function found to be important in this population (22). They were asked “how much do you agree with the following statement … I trust my child’s doctors” and answered “completely agree”, “slightly agree”, or “disagree”. Finally, caregivers were asked “Overall, how satisfied are you with the communication with your doctors and other health professionals?” with response options of “very satisfied”, “fairly satisfied”, or “not at all satisfied”.

*Sources of information* were assessed by asking caregivers “how useful or important each of the following was for you as a source of information … conversations with your medical team (including doctors, psychologists, nurses, social workers)”, “…conversations with doctors or medical providers outside of [Indus or Children’s Hospital]”, “…conversations with your community (for example, with neighbors, community leaders…), “…conversations with your family (siblings, grandparents, aunts, uncles)”, “…conversations with other families at [Indus or Children’s Hospital]”, “…conversations with leaders in your religious or spiritual community”, “…an understanding within yourself (including a feeling, hunch, or dream)”, “…reading in books or looking for information on the internet (e.g. Google)”, and “…social media (e.g. Facebook)”. Response options included “very important”, “a little important”, and “not important” which the participant identified using the visual aid. Sources including conversations with medical providers outside of Indus or Children’s Hospital, as well as conversations with other families and social media were novel for this population. Caregivers were also asked “who do you want to help you understand our child’s diagnosis and treatment?” and selected all that apply from “doctors”, “nurses”, “psychologists”, “pharmacists”, “other”. To further explore communication through the multidisciplinary team, caregivers were asked “How often are you told different things by different members of the medical team, leaving you feeling confused?” and answered “always”, “sometimes”, or “never”. This question was added for the Pakistani context.

*Information preferences* were explored through items including “How important is it to you to know about your child’s likelihood of being cured?” and “How important is it to you to know about how likely it is that cancer or its treatment may affect your child’s life in the future?”. Response options for these questions included “It is very important for me to know…”, “It is not very important for me to know…”, or “I prefer not to know…”. Caregivers were asked “What is your preference for details of information about your child’s diagnosis and treatment?” and answered either “I want to hear as many details as possible in all situations relating to my child’s cancer and it treatment”, “I want to hear details only in certain situations, in other situations I do not want to hear the details”, or “I prefer not to hear a lot of details”. To assess preferences for how information was received, a new question was added asking caregivers “What is your preference for how you receive information about your child’s diagnosis and treatment?” with response options of “I want to hear as much information as possible in my first visit with the doctors and medical team”, “In some situations, I want to hear all of the information at once, and in other situations I want to hear the information over time”, or “I prefer to receive information over time”.

*Experiences with information exchange* were assessed by asking caregivers “How often do you feel like you are given the information that is important to you without needing to ask for it?”, “When you see your child’s doctor, how often do you have questions about your child’s care that you want to discuss but do not?”, and “When you ask questions of your medical team, how often do you get answers that are understandable?”. Response options for all three questions included “always”, “sometimes”, or “never”.

*Caregiver understanding* was further assessed through survey items about their child’s diagnosis and treatment plan. Caregivers were asked open-ended questions including “What is the name of your child’s diagnosis?”, and “Where in your child’s body is the disease located?”. They were asked “Has the disease spread to other places in the body?” with response options of “yes” or “no”. Regarding treatment, caregivers were asked “How long will all of your child’s treatment last?” with response options of “less than 6 months”, “6 months to 1 year”, “more than 1 year, but less than 2 years”, or “2 years or more”. Caregivers selected all that apply for “chemotherapy”, “surgery”, and “radiation” in response to “Which of the following will be part of your child’s cancer?”. Finally, caregivers were asked “What is *your* goal for your child’s treatment?” and “What is your *healthcare team’s* goal for your child’s treatment?” with response options to both questions of “to eliminate my child’s cancer” or “to decrease symptoms from the cancer”.

*Medical information* including the child’s diagnosis, primary tumor location, metastatic disease, and treatment plan (modalities and duration) as well as treatment intent (curative or palliative) were obtained through medical record review.

### Data analysis

Sociodemographic information, communication preferences and experiences, sources of information, and information preferences and experiences were analyzed using descriptive statistics. McNemar’s test was used to assess marginal asymmetry between communication priorities and experiences related to the functions of communication. Chi-square or Fisher’s exact test was used to compare proportions between groups, as appropriate. Responses to questions regarding caregiver understanding of diagnosis, tumor location, metastatic disease, treatment duration, and treatment intent were compared to the information extracted from medical records. For treatment intent, caregiver response of decreased symptoms was considered palliative. For all other items, caregiver responses matching the medical record were considered accurate. A summary variable was created by assigning one point for each correct response about 1) diagnosis, 2) location, 3) metastatic disease, 4) length of treatment, and 5) treatment intent and summing for a total score of 0-5. For caregivers of patients with leukemia, answers regarding metastatic disease were excluded and the score ranged from 0-4. The summary variable was dichotomized into caregivers who gave all accurate responses and those with one or more inaccurate responses. Chi-square or Fisher’s exact tests was used to compare proportions between complete and incomplete understanding in relation to sociodemographic characteristics.

## Results

Study participants included 200 caregivers of children with cancer in Pakistan, including 111 (55.5%) mothers and 55 (27.5%) fathers. Most participants identified as Muslim (96.5%) and reported a monthly household income of <$500 (95.5%). The median patient age was 7 years (range 1-16) and diagnoses included leukemia (67.5%), lymphoma (13.5%), solid tumors (17%), and brain tumors (1.5%). Full demographic information is included in **Table 1**.

**Table 1:** Sociodemographic characteristics.

Characteristic	Overall N = 200*^1^*	Indus Hospital N = 100*^1^*	Children’s Hospital Lahore N = 100*^1^*
Relationship to Patient			
Parent	166 (100%)	73 (44%)	93 (56%)
Other Relative	33 (100%)	26 (79%)	7 (21%)
Missing	1	1	0
Caregiver Gender			
Male	79 (100%)	65 (82%)	14 (18%)
Female	121 (100%)	35 (29%)	86 (71%)
Language			
English	21 (100%)	15 (71%)	6 (29%)
Urdu	119 (100%)	41 (34%)	78 (66%)
Pashto	9 (100%)	8 (89%)	1 (11%)
Other	51 (100%)	36 (71%)	15 (29%)
Ethnicity			
Punjabi	94 (100%)	9 (9.6%)	85 (90%)
Pashtun	17 (100%)	11 (65%)	6 (35%)
Sindhi	27 (100%)	27 (100%)	0 (0%)
Muhajir	33 (100%)	31 (94%)	2 (6.1%)
Baloch	15 (100%)	15 (100%)	0 (0%)
Other	14 (100%)	7 (50%)	7 (50%)
Religion			
Islam	191 (100%)	94 (49%)	97 (51%)
Other Religion	7 (100%)	6 (86%)	1 (14%)
Missing	2	0	2
Province			
Punjab	96 (100%)	5 (5.2%)	91 (95%)
Sindh	70 (100%)	70 (100%)	0 (0%)
Other	34 (100%)	25 (74%)	9 (26%)
Residence location			
Rural (village, small town, district)	83 (100%)	33 (40%)	50 (60%)
Urban	117 (100%)	67 (57%)	50 (43%)
Respondent Education			
No education/Grade 1-5	38 (100%)	18 (47%)	20 (53%)
Grade 6-10	74 (100%)	36 (49%)	38 (51%)
Grade 11-12	41 (100%)	23 (56%)	18 (44%)
Graduate	47 (100%)	23 (49%)	24 (51%)
Monthly Household Income			
$100	121 (100%)	59 (49%)	62 (51%)
$101-$500	69 (100%)	35 (51%)	34 (49%)
$500+	9 (100%)	5 (56%)	4 (44%)
Missing	1	1	0
Distance to Hospital			
<=4 hours	114 (100%)	46 (40%)	68 (60%)
5–12 hours	63 (100%)	32 (51%)	31 (49%)
>12 hours	22 (100%)	21 (95%)	1 (4.5%)
Missing	1	1	0
Number of Children			
<=2	53 (100%)	26 (49%)	27 (51%)
3-4	105 (100%)	49 (47%)	56 (53%)
>4	42 (100%)	25 (60%)	17 (40%)
Electricity			
Yes	195 (100%)	97 (50%)	98 (50%)
No	5 (100%)	3 (60%)	2 (40%)
Water			
Yes	141 (100%)	68 (48%)	73 (52%)
No	54 (100%)	31 (57%)	23 (43%)
Missing	5	1	4
Sanitation			
Yes	158 (100%)	78 (49%)	80 (51%)
No	42 (100%)	22 (52%)	20 (48%)
Patient Age			
0-5	82 (100%)	40 (49%)	42 (51%)
6-10	65 (100%)	31 (48%)	34 (52%)
11-16	53 (100%)	29 (55%)	24 (45%)
Patient Gender			
Boy	128 (100%)	72 (56%)	56 (44%)
Girl	72 (100%)	28 (39%)	44 (61%)
Patient Diagnosis			
Leukemia/Lymphoma	162 (100%)	78 (48%)	84 (52%)
Solid Tumor	34 (100%)	19 (56%)	15 (44%)
Brain Tumor	3 (100%)	3 (100%)	0 (0%)
Histiocytic disorder	1 (100%)	0 (0%)	1 (100%)

*^1^* n (%).

### Communication priorities and experiences

**Figure 1:**
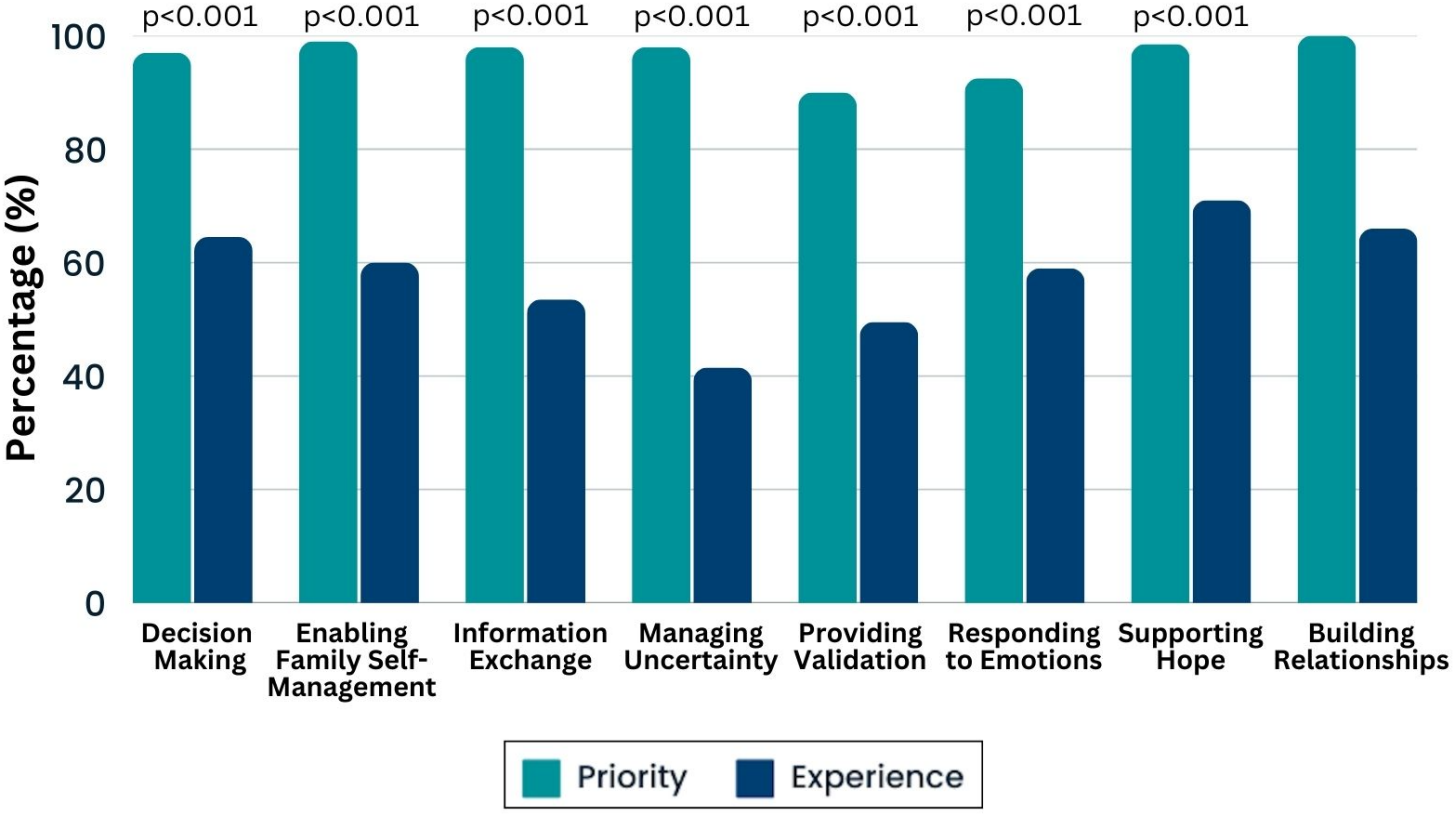
Communication priorities and experiences. Histogram demonstrates the percentage of caregivers who said each priority was “very important” (in teal) and those who experienced this function “most of the time” (blue). McNemar’s test was used to assess marginal asymmetry between priorities and experiences. Since 100% of caregivers rated building relationship as “very important” no comparative test could be utilized.

Most caregivers prioritized all eight functions of pediatric communication with >90% reporting it was “very important to them” that clinicians: “explained things in a way I can understand” (information exchange), “are open and honest with me” (building relationships), “involve me in making decisions about my child’s care” (making decisions), “pay attention to my emotions and feelings” (responding to emotions), “help me deal with the things nobody knows related to my child’s cancer” (managing uncertainty), “help me understand ways to take care of my child while I’m dealing with cancer” (enabling family self-management), “value my thoughts about my child’s health” (providing validation), and “provide me with the information that makes me hopeful about my child’s cancer and treatment” (supporting hope). However, significantly fewer parents (p<0.001) reported experiencing each function (**Figure 1**). Almost all caregivers (96%) completely agreed with the statement “I trust my child’s doctors”. Overall, 67.5% of caregivers were “very satisfied” with communication and 32% were “fairly satisfied”, with only one caregiver “not at all satisfied”.

### Sources of information

**Figure 2:**
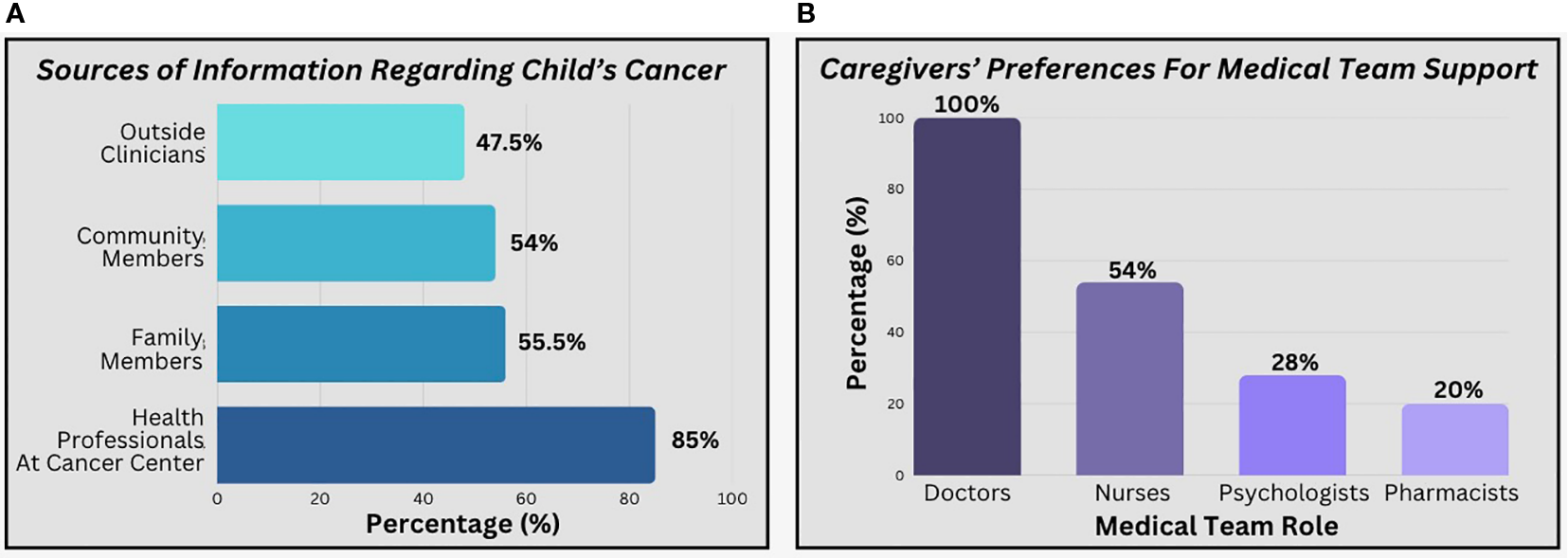
Caregiver preferences regarding sources of information. **(a)** reflects the percentages of caregivers reporting sources of information that were “very important to them”. **(b)** includes percentages of caregivers who selected each profession in a “select all that apply” question regarding who helped them understand diagnosis and treatment.

Caregivers in Pakistan learned about cancer from a variety of sources including community members (54%), family members (55.5%), outside clinicians (47.5%), and health professionals at the cancer center (85%). All caregivers wanted their child’s doctor to help them understand the cancer diagnosis and treatment. However, caregivers also wanted other members of the medical team to help them understand, including nurses (54%), psychologists (28%), and pharmacists (20%). Although they highlighted interprofessional involvement, some caregivers reported that they were “always” (8%) or “sometimes” (56%) told different things by different members of the medical team, leading to confusion. **Figure 2** depicts the various sources of information caregivers reported, including members of the interprofessional health care team.

### Information exchange preferences and experiences

**Figure 3:**
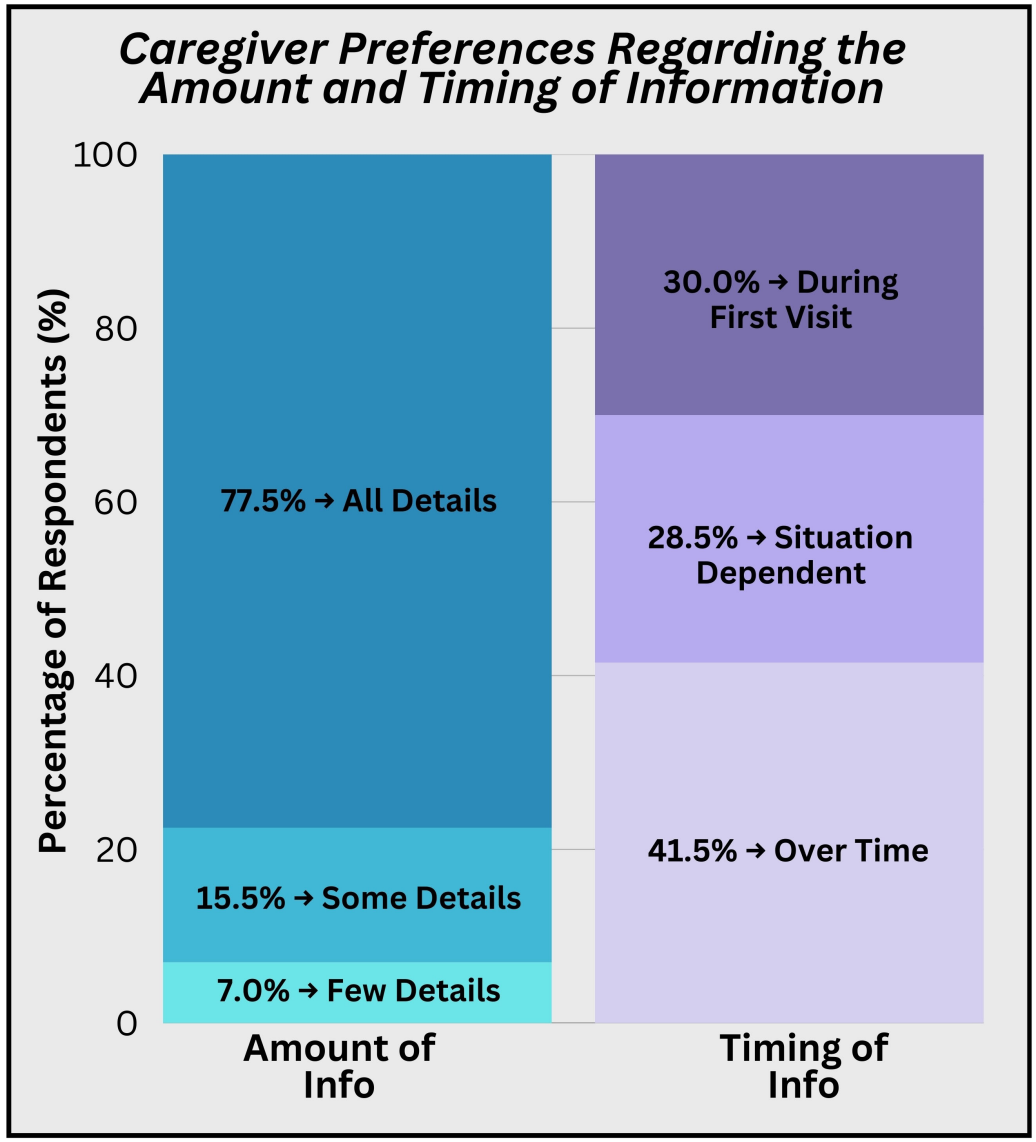
Caregiver preferences regarding timing of information. Caregivers differed in the amount of details they wanted during diagnosis and when they wanted to receive information.

Nearly every caregiver (99.5%) said it was “very important for me to know the likelihood of cure” and 97% said it was “very important for me to know the likelihood this treatment may affect my child in the future”. However, caregivers differed in the way they wanted to receive this information. Many caregivers (77.5%) wanted to hear as many details as possible in all situations, while 15.5% only wanted details in certain situations, and 7% preferred not to hear details. Similarly, there was a range as to whether caregivers wanted as much information as possible upfront (30%), or over time (41%), or differently depending on the situation (28.5%; **Figure 3**).

Only 37.5% of caregivers said they were “always” given the information that was important to them without asking for it, with 48.5% saying “sometimes” and 14% answering “never”. Additionally, many caregivers said they “sometimes” (66.5%) or “always” (12%) had questions about their children’s cancer care that they wanted to discuss with the medical team but did not. When they did ask questions of the medical team, only 59% of caregivers said they “always” got answers that were understandable.

### Caregiver understanding

Comparing medical records to caregiver report, only 76.5% of caregivers correctly understood their child’s diagnosis while 81% correctly understood tumor location. For patients who had diagnoses other than leukemia, 78.5% understood whether the disease was metastatic. Most caregivers (71%) correctly understood the planned length of cancer treatment. Of those who did not correctly identify the length of treatment, almost half (48%) described a shorter length of treatment than outlined in the medical record. Almost all caregivers understood the types of treatment their child would receive including chemotherapy (99% match), surgery (90.5% match), or radiation therapy (90% match). Similarly, nearly all caregivers (96%) correctly understood their child’s treatment intent as curative or non-curative (**Table 2**).

**Table 2:** Caregiver understanding of diagnosis, prognosis, and treatment plan.

Characteristic (N = 200)*	Name of Child’s Diagnosis	Tumor Location	Treatment Intent	Metastatic site (N=65)*	Type of treatment	Chemotherapy	Surgery	Radiation Treatment	Length of Treatment
Match	153 (77%)	162 (81%)	192 (96%)	51 (78%)	169 (85%)	198 (99%)	181 (91%)	180 (90%)	137 (71%)
Mismatch	47 (24%)	38 (19%)	8 (4.0%)	14 (22%)	31 (16%)	2 (1.0%)	19 (9.5%)	20 (10%)	56 (29%)
Missing									7

*Patients with leukemia were excluded from analysis regarding metastatic site.

No associations were found between caregiver understanding and most socio-demographic characteristics including gender, relationship to patient, ethnicity, language, religion, residence, education, income, distance from the hospital, children at home, electricity, water, or sanitation. Similarly, no associations were found between caregiver understanding and patient age or gender. However, caregivers of patients with leukemia had a more comprehensive understanding of their child’s diagnosis and treatment plan (59% all correct) compared to patients with any other diagnosis (18% all correct, p<0.0001; **Table 3**).

**Table 3:** Caregiver understanding by diagnosis type.

Caregiver Understanding by Diagnosis Group (N=200)			
Caregiver Understanding	Diagnosis Group		
	Leukemia	Non-Leukemia	Total
All Correct Matches	80 (59%)	12 (18%)	92
At least 1 incorrect Match	55 (41%)	53 (82%)	108
Total	135	65	200
Missing: 0 (0.00)			
Chi-square Test p-value: <0.0001*			

## Discussion

Our findings provide further evidence for the relevance of the functional model for patient-centered care in diverse populations (13, 23), as all eight functions were highly prioritized by caregivers in Pakistan. Unfortunately, although two-thirds of caregivers were very satisfied with communication overall, gaps were identified between caregiver priorities and experiences, highlighting opportunities for improvement.

Despite only a third of caregivers reporting they received all the information they felt was necessary without asking for it, most endorsed having questions they did not ask their medical team. This may reflect a disempowerment of caregivers, potentially related to medical hierarchy that may be exacerbated in LMICs and particularly in certain cultures or for populations with lower levels of education or health literacy (24, 25). Additional gaps were noted in the objective assessment of information exchange. Interestingly, more caregivers correctly understood treatment intent and type than diagnosis, location, or treatment duration. This may reflect an emphasis by clinicians on treatment plans, including the caregiver’s role in bringing their child to appointments as well as caregiver engagement in everyday care. However, the gap in understanding suggests an opportunity for clinicians in Pakistan to spend additional time explaining diagnosis and potentially using visuals or sharing images to explain tumor location. Caregivers of patients with leukemia were significantly more likely to have complete understanding of their child’s illness. This is consistent with literature from other middle-income countries demonstrating families of children with solid tumors are at particularly high risk for poor information exchange (14). Explanations for this finding were beyond the scope of this study, however it is possible that the relative prevalence of leukemia contributes to increased clinician comfort explaining the disease as well as increased conversation between families being treated for leukemia at each center. Future work should further investigate poor information exchange among patients with diagnoses other than leukemia and focus on interventions to support this population.

Like prior work in other settings (14, 15), almost all caregivers in this study expressed high information needs. However, caregivers expressed variable preferences for how they wanted information conveyed and by whom. It is important that clinicians assess preferences for information exchange, particularly at diagnosis (26), which is an especially vulnerable time in which caregivers may feel overwhelmed or not yet familiar enough with the healthcare system to know which questions to ask. Clinicians must not confuse preferences for delayed information with denial or desire not to receive the information. Notably, many caregivers expressed a desire for interprofessional providers (including doctors, nurses, psychologists, and pharmacists) to be involved in communication, a theme that has been highlighted in literature from high resource settings (27, 28). Particularly in settings where resources are low, it is important for the team to think about how different members might be able to task share and engage in different communication encounters. A risk of team involvement in communication is providing the family with mixed messages. Many caregivers in Pakistan did endorse confusion that arose when different members of the care team shared conflicting information, demonstrating the importance of team communication and strategies including proper documentation (29) and structured handoff (30) to ensure clinicians have a shared understanding of care.

This study should be considered considering its limitations. The study was conducted at only two of the institutions treating children with cancer in Pakistan. While they are the two largest centers in the largest urban areas of the country and were chosen for diversity of funding structure and population served, they may not be representative of all caregivers in Pakistan. Additionally, this study was conducted in two of the many languages and dialects spoken in Pakistan and thus there may have been some selection bias regarding the population captured. Furthermore, the study was conducted at the time of diagnosis (within 8 weeks) to focus on this vulnerable time for communication and minimize recall bias. It was not designed to examine communication priorities and preferences along the cancer continuum and future work should include a longitudinal study or additional cross-sectional work during other important time points. Finally, this study utilized a cross-sectional survey with a 3-point Likert scale. These methods allowed for demonstration of gaps between caregiver priorities and experiences during diagnostic communication as well as various opportunities to improve care but could not fully answer *why* some of these gaps exist. Future work would be needed to test some of the hypotheses presented in this discussion and qualitative methods might foster a better understanding of *why*. Also, although this scale has been used in similarly resourced settings and with other low-literacy populations (17), it is possible that we were unable to capture as much range or nuance as might have been achieved with a 5- or 7-point Likert scale.

In conclusion, caregivers in Pakistan express many of the same priorities for communication as caregivers in other settings and highlight similar gaps in care. Specifically, our results emphasize that all caregivers of children with cancer have high information needs and differ in terms of the details they wish to receive, when, and from whom. This furthers evidence suggesting global pediatric cancer communication can be studied using a shared framework and emphasizes that clinicians and caregivers of children with cancer around the world face many of the same communication challenges. Interventional work should focus on maximizing human resources through interprofessional communication as well as ensuring complete diagnostic information is conveyed during initial visits, particularly for families of patients with diagnoses other than leukemia, and that caregivers are repeatedly empowered and encouraged to ask questions of their care team.

## Data Availability

The raw data supporting the conclusions of this article will be made available by the authors, without undue reservation.
